# Comparing Feedback Types in Multimedia Learning of Speech by Young Children With Common Speech Sound Disorders: Research Protocol for a Pretest Posttest Independent Measures Control Trial

**DOI:** 10.3389/fpsyg.2018.00444

**Published:** 2018-04-05

**Authors:** Wendy Doubé, Paul Carding, Kieran Flanagan, Jordy Kaufman, Hannah Armitage

**Affiliations:** ^1^Department of Film and Animation, Faculty of Health Arts and Design, Swinburne University of Technology, Melbourne, VIC, Australia; ^2^Speech Pathology, Australian Catholic University, Brisbane, QLD, Australia; ^3^Department of Psychological Sciences, Faculty of Health Arts and Design, Swinburne University of Technology, Melbourne, VIC, Australia

**Keywords:** feedback, speech sound disorder, phonological disorder, multimedia learning, video game, mobile application

## Abstract

Children with speech sound disorders benefit from feedback about the accuracy of sounds they make. Home practice can reinforce feedback received from speech pathologists. Games in mobile device applications could encourage home practice, but those currently available are of limited value because they are unlikely to elaborate “Correct”/”Incorrect” feedback with information that can assist in improving the accuracy of the sound. This protocol proposes a “Wizard of Oz” experiment that aims to provide evidence for the provision of effective multimedia feedback for speech sound development. Children with two common speech sound disorders will play a game on a mobile device and make speech sounds when prompted by the game. A human “Wizard” will provide feedback on the accuracy of the sound but the children will perceive the feedback as coming from the game. Groups of 30 young children will be randomly allocated to one of five conditions: four types of feedback and a control which does not play the game. The results of this experiment will inform not only speech sound therapy, but also other types of language learning, both in general, and in multimedia applications. This experiment is a cost-effective precursor to the development of a mobile application that employs pedagogically and clinically sound processes for speech development in young children.

## Introduction

Speech development resources and materials for use on tablet-based and touchscreen devices have become widely available in recent years. However, these resources are severely restricted by the lack of research knowledge about how best to get children to engage, interact and learn from their use. In clinical and classroom settings children with significant speech sound disorders require highly individualized feedback that takes into consideration the child's diagnosis, performance and personal factors (e.g., motivation, attention and self-esteem). Outside the clinic and the classroom, they receive feedback from interaction with the world around them. In particular, speech pathology treatments benefit from home practice. However, applications for mobile devices are unlikely to provide feedback on speech sounds produced by children (see section Preliminary App Search). This project will set up a series of experiments (The Wizard of Oz experiments) to systematically examine which aspects of feedback are most effective in engaging and motivating children with speech sound disorders to use the computer technology that is currently available. This will ultimately result in developed portable technologies which will allow children with speech sound disorders to fully engage and benefit from highly sophisticated speech training “apps” on tablet, web and smart phone devices to assist with homework when engaged in speech pathology interventions with a speech pathologist and consequently develop intelligible and age-appropriate speech. This technology will provide much needed resources for large numbers of children who live in regional, remote and rural settings and who have very limited access to specialist remediation and treatment.

By far, the two most common speech sound disorders are “phonological delay” and “phonological disorder” with approximately 75% of children with a speech sound disorder meeting the criteria for these diagnoses (Dodd, 2005). Children with phonological delay/disorder are able to produce all sounds expected for their age but make systematic sound substitutions that are either typical of younger children (in the case of phonological delay) or not seen in typical development (in the case of phonological disorder) (Dodd, 2014). For example, a child with a phonological delay may have the error pattern of “stopping” where all fricative sounds (e.g., /s/, /f/, /v/, /z/ etc) are produced as stops (e.g., /t/, /p/, /b/, /d/ etc.) such that “sun” would be produced as “tun” and “fan” as “pan.” Although theories of phonological development vary, it is widely held that minimal pair therapy in the context of speech pathology intervention can be used to resolve phonological delay and disorder (Baker, 2010). In most iterations of the technique, minimal pair therapy involves presenting a child with word pairs that differ by one minimal feature so that the child's error pattern would cause the two words to sound the same (i.e., as homophones). Using the aforementioned example of the child producing fricatives as stops, the child would produce the words “see” and “tea” as “tea” or “four” and “paw” as “paw,” “zoo” and “do” as “do” etc. In the context of therapy, the child's error of producing the minimal pair targets as homophones (e.g., “see” and “tea” as “tea”) creates semantic confusion for the treating clinician and, as a result, the clinician provides feedback to the child that both productions sound the same (e.g., all productions produced as “tea”). This feedback encourages the child to make a phonetic contrast between the minimal pair words (e.g., contrast between the fricative /s/ and stop /t/ sounds in “sea” and “tea”) so that the clinician can distinguish between the child's production of the minimal pair words (Weiner and Ostrowski, 1979). Quite apart from theories of phonological development and representation, the effectiveness of phonological therapy is likely to derive from feedback from the clinician of the child's homophonic productions and the child's motivation to rectify this error.

### Feedback in a multimedia environment

Feedback can be defined as “any message that is generated in response to a student's action” (Mason and Bruning, 2001). Feedback is a vital component of learning (Laurillard, 2002; Shute, 2008; Butler et al., 2013) and the motivational processes that support it (Harks et al., 2014) in both face-to-face and computer-based environments (Mason and Bruning, 2001; Moreno, 2004; Corbalan et al., 2009). The terminology for types of feedback is not consistent. For consistency and for relevance to this investigation of both multimedia learning and speech therapy, this study predominantly adopts terminology and concepts gleaned from “a large body of research” in Narciss et al. (2014) largely consistent with the comprehensive literature review of factors influencing formative feedback in Shute (2008) which includes feedback categories specifically for multimedia discussed in Narciss and Huth (2004). See Table 1 for the sources of each category in the taxonomy we adopted which we will now describe. Systematic reviews of feedback in learning suggest that, notwithstanding instructional, individual, and situational differences, feedback is generally more effective when elaborated with an informational, or *formative*, component (Mason and Bruning, 2001; Shute, 2008; Narciss et al., 2014). In contrast to formative feedback, *summative* feedback of a mark or grade, and *verification* feedback indicating the correctness of a learner response, do not contain information to assist learners in improving their understanding.

**Table 1:** Types of feedback commonly used to teach sounds and suitable for multimedia environments, with categories in decreasing order of complexity.

**Feedback category**	**Type of formative and verification feedback**	**Description**
Intrinsic (Laurillard, 2002)		Intrinsic occurs directly in response to a learner action but is not a comment on the action.
Extrinsic (Laurillard, 2002)		A comment on a learner response or action. Formative and Verification feedback are Extrinsic.
Formative (Shute, 2008; Narciss et al., 2014)		Contains Information to assist the learner in bridging the gap between their conception and the concepts to be learned. Can be topic contingent or response contingent
Topic contingent (Mason and Bruning, 2001; Shute, 2008; Narciss et al., 2014)		Elaborative information about the topic being learned. Shute suggests this can reteach the same material whereas Mason and Narciss describe directing learners to search for information themselves.
Response contingent (Shute, 2008)		Elaborative information about the learner's response explaining reasons for both incorrect and correct responses.
	KP (Narciss et al., 2014)	Knowledge about how to Process the task addresses procedural knowledge. It can be either topic or response contingent.
	KM (Shute, 2008; Narciss et al., 2014)	Knowledge of Mistakes finds errors or provides hints for finding them. Can be either topic or response contingent. Also known as Bugs/Misconceptions when response contingent.
	KC (Narciss et al., 2014)	Knowledge of Concepts is topic contingent information about concepts.
Verification (Shute, 2008; Narciss et al., 2014)		Does not contain information other than correct / incorrect.
	KCR(Shute, 2008)	Knowledge of Correct Response describes correct answer but no other information.
	RUC(Shute, 2008)	Repeat Until Correct indicates incorrect by presenting the task again and proceeds to the next task when a correct response is received. It provides no other information. Also known as Try Again (Shute) or Answer until Correct (Narciss).
	MT (Shute, 2008; Narciss et al., 2014)	Multiple Try proceeds to the next task when a correct response is received or after a predetermined limited number of attempts.
	KR (Shute, 2008)	Knowledge of Results indicates correct or incorrect but provides no other information.

In this paragraph we draw upon examples from speech pathology approaches to illustrate the taxonomy of feedback terminology we adopted, roughly in order of increasing complexity. When working with a child with speech sound disorder (such as a phonological disorder) speech pathologists might highlight errors by claiming to misunderstand the child (Yont et al., 2000; Masso et al., 2014). When the speech pathologist provides no other feedback or cues to assist in correcting the error, this approach could be categorized as *verification* feedback. The verification feedback would be classified as *Knowledge of Response* (KR) when the lesson immediately moves on to a new sound or *Multiple Try* (MT) when the child can make a finite number of attempts or *Repeat Until Correct* (RUC) when the child continues making sounds until an acceptable sound is produced. If the error persists, a speech pathologist might present the sound/word again, providing *Knowledge of Correct Response* (KCR) (e.g., “Try and say “see”). In the absence of speech production literature, Maas et al. (2008) extrapolated from sensorimotor learning literature to suggest that KR alone may be insufficient for learners, who, in the early stages of speech therapy may require further instruction about the processes in achieving an accurate production or may have difficulty discriminating between a correct or incorrect production. Similarly, Hewlet (1990) postulated that children need, in addition to awareness of errors in production, knowledge of the ways to articulate a speech target. In other words, *formative* feedback could be more effective than the types of *verification* feedback described above. A demonstration or description of the articulatory processes associated with the sound is a type of formative feedback described as *Knowledge of How to Process the Task* (KP) (Narciss et al., 2014). In this case, the speech pathologist might supplement the KR or KCR with formative feedback providing information about the task to be performed, the underlying concepts or topics in producing the sound (e.g., “Make sure you use your “long sound” when you say “see”), or the processes in producing the sound (e.g., “To make your long sound /s/, make sure that you put your tongue behind your teeth and make long, gentle air”). If the feedback explains the difference between the child's articulatory processes (*Knowledge of Mistake, KM*) and the correct articulation, it would be *response contingent* (e.g., “You said “tea” with a short sound. You need to say “see” with a long sound). If it explains the correct processes without referring to the child's processes, it would be *topic contingent* (e.g., “You need to use your long sound”). Although response contingent feedback is generally considered more effective than topic contingent feedback, procedural skills have been found to benefit from KCR followed by topic contingent feedback, without reference to errors (Shute, 2008). From a narrative review of computer-based instruction, Mason and Bruning (2001) present a model of feedback variables that suggests that KCR and response contingent feedback is suitable in low level tasks for learners with low levels of prior knowledge whereas KCR followed by topic contingent feedback is more suitable for learners with higher levels of prior knowledge.

In this paragraph we discuss feedback in multimedia environments. Children initially learn to speak when interacting with their carers and the wider world (Topping et al., 2013). A multimedia environment could simulate natural settings for speech production and language development within mobile device applications thus providing additional opportunities for practice which can build on children's experiences. Cognitive Theory of Multimedia Learning (CTML) provides an evidence-based framework for learning with visual and auditory sensory channels (Mayer, 2005). CTML has been extended to game-play (Mayer, 2014a) and E-learning (Clark and Mayer, 2016) and can consequently guide the development of a virtual speech therapist. Although CTML examines interactivity, it does not focus on feedback. In contrast, the Interactive Tutoring Feedback model views feedback as “one of several basic components of a generic feedback loop, not as an isolated element of instruction” (Narciss, 2017). Similarly, the Conversational Framework for Multimedia Learning (Laurillard, 2002) asserts that multimedia learning environments should attempt to replicate learner-teacher dialogue, repeating the feedback loop until the learner's conception matches that of the instructor. Within Laurillard's framework, *intrinsic* feedback received directly in response to learner action on the multimedia environment can assist in relating the learner's concrete experiences of the world to learning goals whereas *extrinsic* feedback is received as a comment on the learner action and would require additional cognitive processing (Laurillard, 2002, pp. 58–69). In natural settings children would receive intrinsic feedback on the clarity of their speech from responses to their questions or requests, for example, if they ask for more meat but receive a glass of milk instead. Furthermore, “. although not an inevitable response to the action, [correction of] pronunciation is a social norm and feedback of this type is natural and probable in a social situation” (Laurillard, 2002, p. 62). An example of intrinsic feedback in a multimedia environment would be an object such as a spaceship moving fast in response to a child saying “fast” or moving slowly when the child says “slow.” Most summative, verification and formative feedback discussed in the paragraph above can be thought of as extrinsic. It could be argued that the speech pathology approach of semantic confusion used as feedback in minimal pair therapy is not merely KR but is instead a type of intrinsic feedback that mirrors a real-world response.

Table 1 summarizes our discussion of types of feedback commonly used in speech pathology sessions and suitable for multimedia environments. It illustrates the hierarchy from the postulated highest level of complexity (intrinsic) to the lowest level (verification). To assist the reader, Table 2 presents some further examples of multimedia implementations of the individual type of feedback in response to speech sound pronunciation by children.

**Table 2:** Examples of implementation in multimedia for common types of feedback.

**Type of feedback**	**Multimedia example**	**Multimedia feedback example**
KR/RUC	Memory card game A grid of cards is presented face down. The card faces consist of pairs of pictures. The child: •Taps on a card to see a picture and hear the word for that picture •Taps on another card to see the matching picture •Says the word for the picture If the correct picture is chosen and the word is correctly pronounced, the child's score is increased by 1 and the pair is removed from the grid. In multiplayer games, the winner has the greatest number of pairs.	If the word is not pronounced correctly, KR–The pair of cards returns to face down. In multiplayer games another player takes a turn RUC–If the picture matches, the child can say the word in the picture again until correct, or MT–for a predetermined number of attempts (for example, three).
Intrinsic	Adventure video game Objects in a game perform actions in response to accurate pronunciation. For example, a character moves from one scene to another, collecting or using objects along the way to help their journey. A gate object is highlighted in the game, and the child hears the word “gate.” The child then says the word “gate.”	If the word is not pronounced correctly, the gate remains closed. In minimal pairs, if the child makes the predicted error, the image of a gate could be replaced with an image of a date (the predicted error). If it is correctly pronounced, the gate opens and the next scene appears on the screen. If the word is partially correct, the gate opens partly, in response to the degree of correctness, but the next screen does not appear.
KP	3D Talking head and/or elaborative feedback comments	The talking head demonstrates correct movements. Text or spoken comments such as “Move your tongue further back in your mouth” could be presented either alone or accompanying the talking head.

Limited evidence can be found in literature reviews for the ability of educational computer games to support the acquisition of knowledge and skills (Connolly et al., 2012; Merchant et al., 2014; Boyle et al., 2016; Clark and Mayer, 2016, pp. 368–389; Hainey et al., 2016). Importantly, strong evidence can be found of an association between elaborative or formative feedback and positive learning outcomes from computer games, with feedback type depending on a range of variables such as learning content and learner prior knowledge (Moreno, 2004; Merchant et al., 2014). The inclusion of motivational features such as autonomy, appealing colors and face-like images can support learning if they comply with CTML principles, in particular when relevant to the content and not a source of distraction (Ryan et al., 2006; Mayer, 2014b). Strong evidence of positive learning outcomes supports personalization, in particular with avatars which gesture and speak polite conversational language with a human voice (Clark and Mayer, 2016, pp. 169–200).

### Wizard of Oz experiments

**Figure 1:**
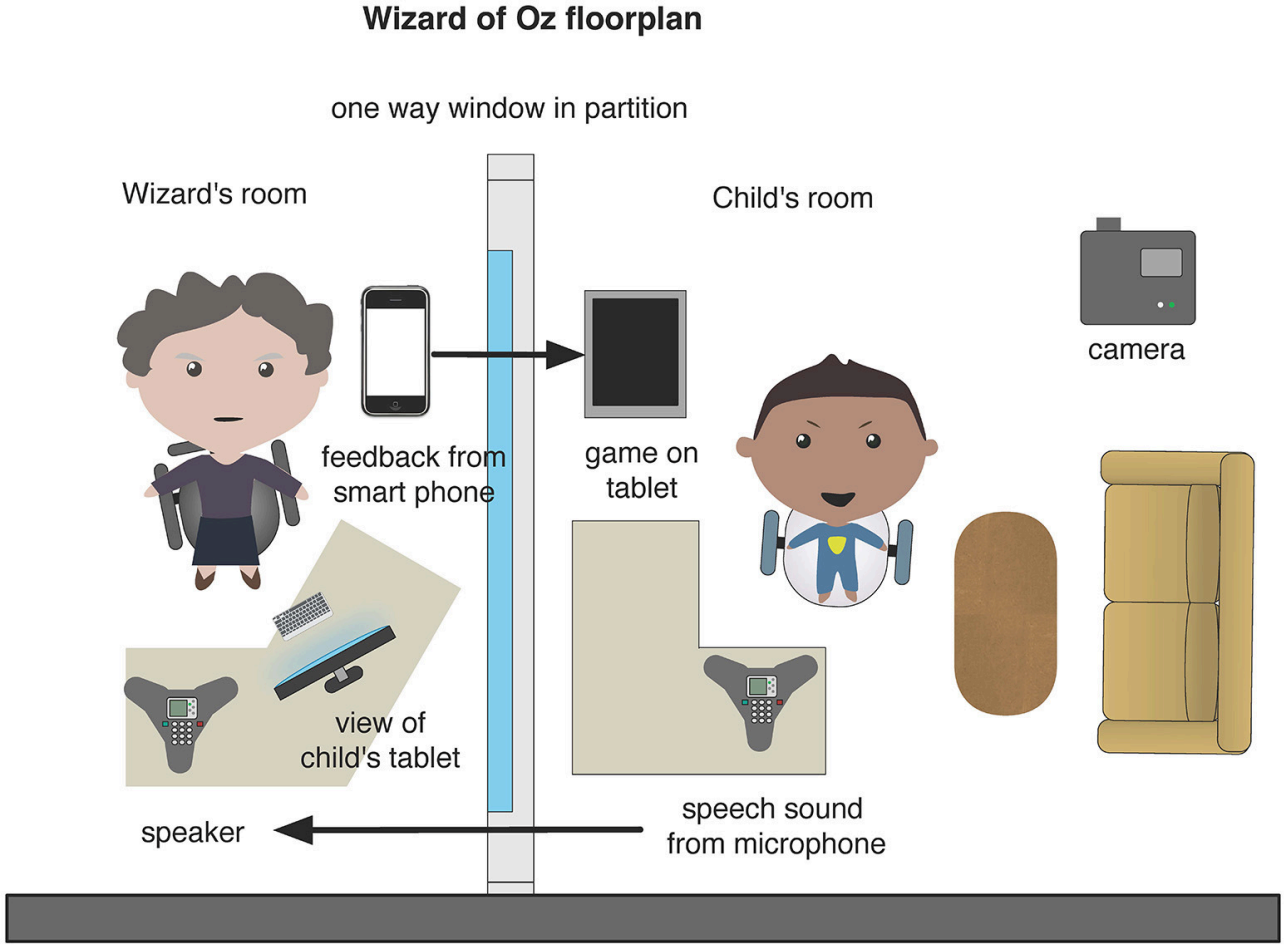
“Wizard of Oz” setup.

**Figure 2:**
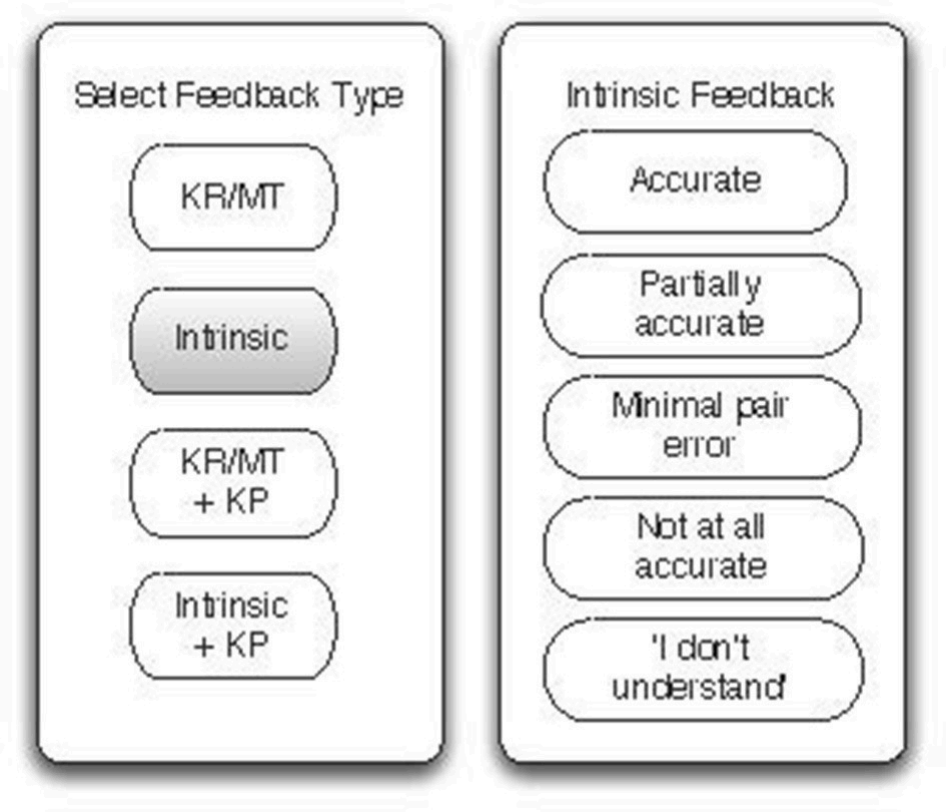
The Main menu screen and the Intrinsic feedback menu screen seen by the “Wizard”.

Speech pathology applications for independent practice could benefit from speech pathology and multimedia learning expertise during their design and before the expense of development. “Wizard of Oz” experiments provide learners with a computer interface, but supply feedback from a hidden human expert as illustrated in Figures 1, 2. The approach was used to influence the design of feedback for adult second language learners (Engwall et al., 2006; Engwall and Bälter, 2008) but has not been used to investigate speech sound development to our knowledge. We envisage that a “Wizard of Oz” experiment would provide evidence for the provision of effective multimedia feedback for speech sound development.

### Aims and objectives

This study is the first step toward our long-term objective to create a Virtual Speech Therapist that employs pedagogically and clinically sound processes for speech development in young children to assist with home practice as part of therapy with a speech pathologist. Automatic speech recognition (ASR) systems are complex and time-consuming to develop. Sophisticated algorithms are required to cover all possible pronunciations, correct and incorrect, in different voices and background noise conditions (Renals and King, 2010). In speech and language learning, they may identify correct pronunciation as incorrect and vice-versa (Massaro and Light, 2004; van Doremalen et al., 2016). Our study will investigate the effectiveness of different types of feedback for speech sound substitutions occurring in phonological disorders, unhindered by confounding problems in automatic speech recognition (Massaro and Light, 2004) and before committing resources to the development of complicated software.

This study aims to employ a “Wizard of Oz” design to evaluate the efficacy within a multimedia platform of four types of feedback to assist speech development in young children. In particular, the study aims to compare predominant types of feedback given by speech pathologists during minimal pair therapy, and those recommended in learning literature, with those recommended for multimedia learning.

### Research questions

1. How effective are different types of feedback in improving speech sounds in young children while using a multimedia platform?
2. What variables influence the effectiveness of each type of feedback?

## Materials and equipment

### Participants

Children aged between 4 and 6 with phonological delay or phonological disorder as identified by a qualified speech pathologist using the Developmental Evaluation of Articulation and Phonology (DEAP; Dodd et al., 2002) will be randomly allocated to five groups, each with 30 participants. Phonological delay is defined as the consistent use of phonological processes that are used in typical development that are supressed by 90% of children of the same chronological age (Dodd, 2014). Phonological disorder was defined as the consistent use of phonological processes in which some of the phonological processes used by that child are not used by 90% of children at any stage of their development (Dodd, 2014). The children will all consistently use one or more of the three phonological processes to be targeted by the intervention of this study (stopping, fronting or cluster reduction). The severity of each child's diagnosis will be either mild, moderate or severe with reference to the normative data for percentage consonants correct (PCC) as measured by the DEAP.

All children will be monolingual English speakers with receptive language within or above the expected range for their age as measured by the Clinical Evaluation of Language Fundamentals –Preschool 2nd Edition (Wiig et al., 2006) or Clinical Evaluation of Language Fundamentals –Fourth Edition (Semel et al., 2006). Children will have hearing skills within normal limits as measured by their last hearing test within the last 12 months and will not have other symptoms of developmental delay as reported by parents. The children will also have no apparent deficits in structure and function in oromotor structure and function as determined by examination by a speech pathologist.

### Design

**Figure 3:**
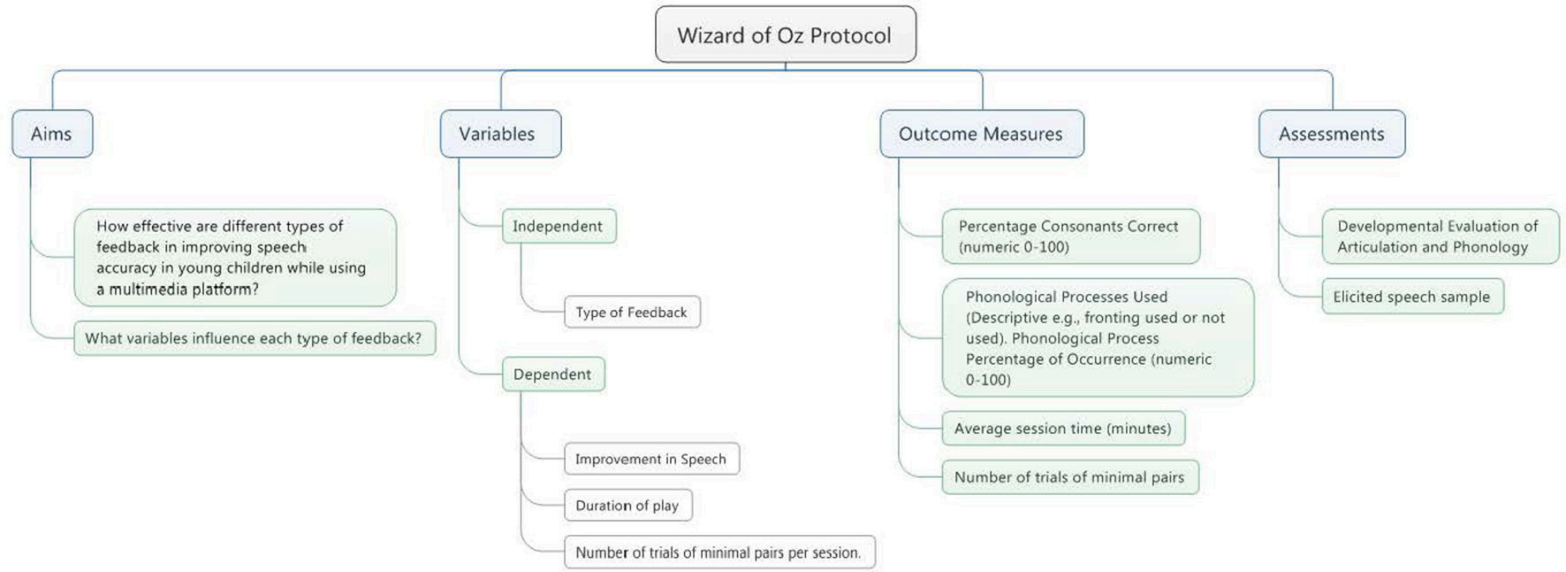
Study design.

The study will be a pretest posttest independent measures design with a five level experiment consisting of a control group and a 2 × 2 design for type of feedback (intrinsic or KR) and presence of topic contingent KP feedback in the form of articulation guidance. See Figure 3 for an overview of the aims, variables, outcomes and measures employed in this experiment.

Children will be randomly allocated to one of four experimental groups or to a control group. For type of feedback, verification feedback types KR/MT/RUC are not highly recommended because they do not supply information to guide the learner. However, KR/MT will be included because it is prevalent in multimedia applications, mainly because it is easy to implement. RUC will be excluded because the number of attempts cannot be controlled and because it may inhibit the timely progress of some children. Intrinsic feedback is recommended for multimedia applications because it can assist learning by building upon, and relating to learners' real-world experience (Laurillard, 2002, pp. 58–69; Ke, 2014). We are interested in comparing these two types of feedback in an attempt to verify the value of the less easily implemented intrinsic feedback. KP will also be included because it is arguably the most effective type of formative feedback (Shute, 2008) and also because it is prevalent in speech pathology practice (e.g., Maas et al., 2008). Whereas, KR/MT and intrinsic feedback can be provided alone or in combination with other types of feedback, KP usually follows and elaborates another type of feedback. Consequently, two types of feedback (KR/MT and Intrinsic) will be provided in isolation to highlight their differences and a further two will combine them with KP. The combined KR and KP condition will allow us to more closely simulate speech pathology sessions whereas the combined intrinsic and KP condition is recommended as best practice for multimedia environments. A fifth condition will act as a control for changes in speech development which could occur over time, either naturally or because of other interventions. KCR will not be provided as feedback because it will appear as a prompt when the sound is presented to the child. Table 3 summarizes the rationale behind the choices of types of feedback to be compared in the study. Table 4 presents examples of feedback provided to each experimental group.

**Table 3:** Rationale for feedback type in experimental conditions.

**Group**	**Rationale for use of type of feedback in treatment**	**Feedback type**
1	Prevalent in multimedia applications for speech production because it is easy to implement.	KR/MT
2	Simulates natural settings. Recommended in evidence-based literature for learning with multimedia. Included to explore if learning benefits outweigh development costs.	Intrinsic
3	Recommended in learning literature. Prevalent in speech pathology sessions for young children with speech sound disorders.	KR/MT + topic contingent KP
4	Recommended as best practice in evidence-based literature for learning with multimedia.	Intrinsic + topic contingent KP

**Table 4:** Context of presentation and feedback delivery for each group.

**Group**	**Feedback type**	**Context**	**Feedback delivery**	
			Correct	Incorrect
1–4		Basic cue–the avatar speaks a word and an image of the word is presented.		
1	KR/MT	Basic	“That's right. Let's move on to the next word.”	“Try again.” After three attempts, “Not quite but let's try another word.”
2	Intrinsic	Basic + Image becomes an interactive object in a game. Avatar says “can you make …. happen when you say ….?”	The interaction happens in a way that indicates the child's utterance has been understood and the game proceeds to the next interaction.	If the child's utterance is: (a) Not at all accurate, the object is haloed and no action occurs. (b) Not accurate but partially understandable, the object is haloed, a partial action occurs, but the object returns to its base state. (c) The predicted minimal pair error, the object is shown as the pair and then returns to its base state. After three attempts the next object is presented. Possibly comment “I don't understand what you said”
3	KR/MT +KP	As in 1	As in 1	As in 1 plus a spoken comment describing correct articulation
4	Intrinsic +KP	As in 2	As in 2	As in 2 plus a spoken comment describing correct articulation
5	Control group	N/A	N/A	N/A

### Outcome measurement

The relative benefits of the different experimental conditions will be measured by the primary measure of changes in use of phonological processes and PCC from pre-test to post-test as measured by the DEAP (Dodd et al., 2002). Such assessment will capture suppression of the phonological processes targeted in treatment and generalization effects (if any) to other phonological processes. Phonological process percentage of occurance (Randolph and Wendt, 2014; i.e., the number of times that a child uses a given phonological process) will also be measured and compared pre and posttest. Pre and post-test assessments will be recorded and a sample of the assessments independently analyzed followed by interrater reliability checks. In addition to the pre-test/post-test measures, number of trials and percentage accuracy of trials per session (cumulative accuracy) will be measured for each experimental condition as further indication of the effectiveness of feedback of that condition and determine when goals of intervention have been reached. For example, if a child reaches 90% accuracy of production of target words for one phonological process, another will be targeted. It could be argued that motivation to continue playing will increase practice and consequently improve outcomes (Williams, 2012). A secondary measure of motivation as a variable that could influence the effectiveness of feedback type will be the amount of time participants choose to continue playing the game (Deci et al., 1999) up to a total of 20 min as well as the number of trials they complete.

### Stimuli

Targets for intervention will be minimal pairs addressing the phonological processes of stopping, velar fronting and cluster reduction as, according to Dodd et al. (2002) 90% of children over 3;11 (3 years and 11 months of age) will have suppressed these phonological processes and they are therefore considered errors at this stage.

The target words comprising the minimal pairs will be:

- High frequency, high imageability words
- Exclude the consonants /∫/ (“sh”), /θ/ (voiceless “th”) and /ð/ (voiced “th”) as, according to Dodd et al. (2002) 90% of children by the age of 4;0 can produce all consonants except these.
- Exclude words with triclusters (i.e., words with three sounds in a consonant cluster such as “splash”)
- Initial word position as people tend to identify initial sounds more readily than medial or final sounds (Redford and Diehl, 1999).

For each phonological process addressed, five different examples for each minimal pair will be included as stimuli in the experimental tasks. For example, an experimental task targeting stopping could include the minimal pairs “fin-pin,” “see-tea,” “zoo-do,” “fat-pat,” and “zip-dip.” Hodson (2010) suggested that at least two exemplars of each phonological process be targeted in therapy. In total, 15 minimal pairs will be addressed across the experimental activities. All minimal pairs will be matched for age of acquisition, imageability and concreteness based on the MRC Psycholinguistic database and frequency using the SUBTLEX-UK database (van Heuven et al., 2014).

### Wizard of Oz setup

The “Wizard of Oz” application will be designed according to CTML principles (Mayer, 2014a,b; Clark and Mayer, 2016). The experiment will take place in the Swinburne University of Technology BabyLab which has rooms configured as per Figure 1 with microphones, speakers, cameras, desktop computers and one way windows. The speech pathologist “Wizard” will be hidden from the child. The child will play the game on a mobile tablet device and produce speech sounds as part of the game. The child's speech sounds will be transmitted to a speaker in the Wizard's room. The Wizard will see a range of feedback options on a mobile device such as a smart phone (Figure 2) and select the appropriate option which will immediately take effect on the child's tablet. Information captured by video camera and screen capture will be available for analysis.

### Intervention procedure

Each child in the four experimental groups will see and hear a word as spoken by an avatar in the application on their tablet device. The avatar will ask the child to repeat the word. As shown in Figure 1 the “Wizard” will hear the child's response and view a monitor display of the child's screen. The “Wizard” will also see each feedback option on a mobile device and will thus be enabled to immediately assess the utterance and select the appropriate feedback. The child will then see and/or hear the selected feedback as delivered by the avatar in the application.

Each experimental group will receive the same set of words and the same basic presentation of an animal avatar which speaks polite conversational language in a human voice. The words will be presented in random order, rather than a predetermined sequence, to enable children to keep playing as long as they choose. The experimental activity is anticipated to take approximately 10 min. Based on other similar studies in the same laboratory (e.g., Huber et al., 2016) it is not anticipated that most children will chose to play for longer than 20 min. Each word will be spoken by the avatar, simultaneously as it is presented as an image. The avatar will then prompt the child to say the word. The treatment for each group will differ in the context of the presentation and in the feedback delivery as shown Table 4.

### Session schedule

Dosage is a significant variable in intervention for speech sound disorders (Baker, 2012; Kaipa and Peterson, 2016) and relates to the number of treatment sessions, the frequency of treatment sessions, length of treatment sessions and number of trials per treatment session (Baker, 2012). A clear precedent has not been set in the literature as to the most effective or efficacious dosage for phonological therapy, let alone home practice (which the study is aiming to investigate). There is evidence to suggest that treatment should occur three times per week (Allen, 2013) but daily home practice is also used (Crosbie et al., 2005). The number of sessions rarely exceeds 16–21 sessions in total in studies of phonological therapy (Crosbie et al., 2005; Williams, 2012). Despite the novelty of the study in exploring practice with an app the above literature will be used as a guide such that each participant will attend the Swinburne Babylab facility for individual sessions 3–4 days per week over a 2–4 week period for a total of 12 sessions (in addition to testing sessions). As the study seeks to investigate the number of trials (from data on percentage accuracy per trial) and length of session as measures of effectiveness of feedback and motivation, respectively, these parameters of dosage will not be set but we expect that children will engage for up to 20 min completing 80–100 trials.

**Figure 4:**
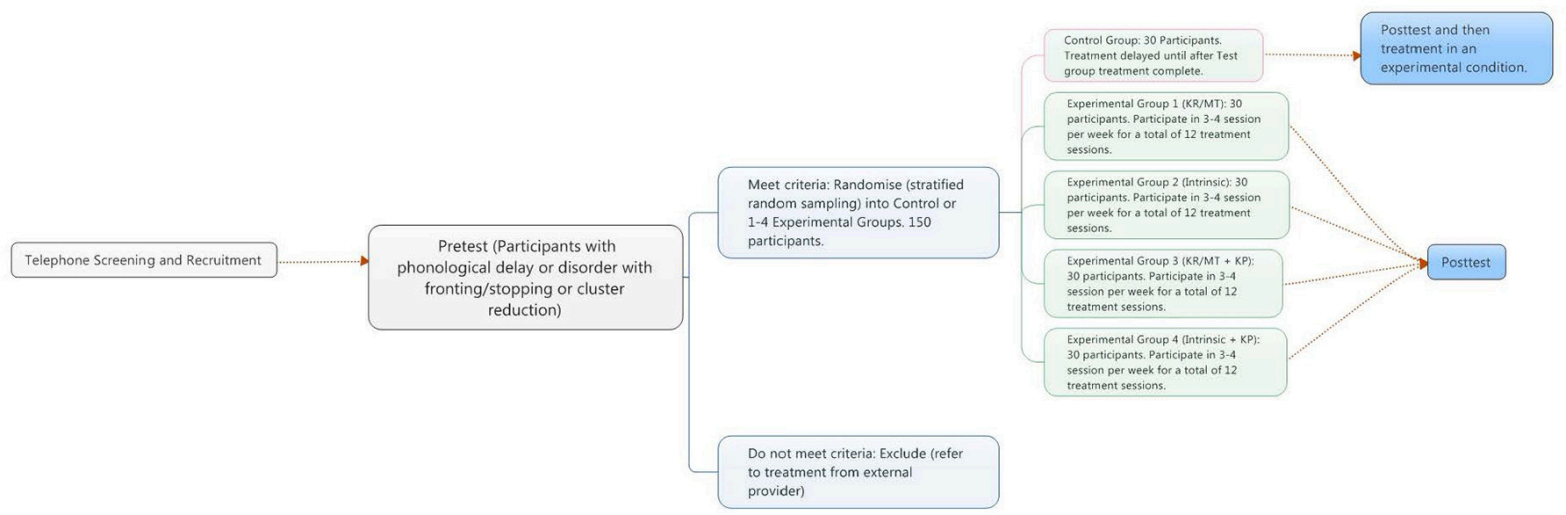
Session schedule.

At the start of the first session children will be evaluated by a qualified speech pathologist with experience in the assessment of children with speech sound disorders to confirm that they fit within the inclusion criteria. This step will be accomplished by collecting a case history and include questions relating to the inclusionary and exclusionary questions as well as previous exposure to speech pathology intervention and speech pathology apps. During this assessment session, the speech pathologist will work through a checklist of instructions which are exemplary of instructions used in the therapy task to ascertain the stimulability of the therapy targets and that the child's comprehension of the instructions to be used in the experimental tasks. Errors in comprehension will be corrected by the speech pathologist within this session. Participants will be randomly assigned to a feedback condition or to the control condition and then take the pre-test. The pre-test and post-test will be offered in a room separate from the Babylab to reduce encoding specificity effects that would provide an advantage to the children in the feedback condition relative to those in the control condition. The control group will attend the first session and then return between 2 and 4 weeks later to take posttest. After completing the pretest, the experimental group will be offered 12 sessions of the experimental activities over a 4-week period. The experimental groups will then be familiarized with the Babylab. Each of sessions two to 12 will consist of approximately 10 min for the experimental task followed by approximately 10 min of an unrelated game or activity for an expected total of 20 min. Children will determine when they want to stop playing the experimental activity. As stated above, the children will complete 80–100 trials of the target minimal pairs but the exact number of trials completed by each child for each session will be recorded and checked for equity between participants. On completion of the experimental task in session thirteen, children will move from the Babylab to take the post-test. See Figure 4 for a diagram of the session schedule.

## Stepwise procedures

### Preliminary app search

In August 2016 we conducted a preliminary search of unbundled speech therapy applications currently available on the iTunes store for the learning of English speech sounds by children aged 5 years of age and younger. None of the 44 apps returned by the search supplied feedback for the child's utterance. Nineteen apps relied entirely upon speech pathologists and others to provide feedback. Three apps provided feedback specifically to supplement human feedback. Feedback provided by 25 apps was predominantly verification feedback, which requires less informed judgment of the correctness of the utterance compared with elaborative feedback and consequently requires less complicated software. Fifteen apps provided KR feedback alone, and a further four provided KR in combination with other types of feedback. In summary, from the perspective of the literature discussed in this protocol, the apps returned by the search provide insufficient feedback to be of value during independent practice.

### Ethics

The protocol was approved by Swinburne University Human Ethics Research Committee: SHR Project 2017/262 on October 20 2017, in accordance with the National Statement on Ethical Conduct in Human Research (2007) and the Australian Code for the Responsible Conduct of Research. The assent of children participating will be sought as well as the consent of a parent or legal guardian as per the NHMRC National Statement Guideline 4.2.7.

The primary operational concern in conducting research into speech sound disorders is the ethical concern of using a non-standard or tested treatment with children. Speech pathology treatments should be characterized by beneficence and non-maleficence yet a non-standard treatment may be of no benefit and delay a child's access to beneficial treatment. The trial described in this protocol avoids these ethical concerns for several reasons. Firstly, the length of this trial is only 3 weeks which is shorter than average waiting list times for speech pathology services (Community Affairs References Committee, 2014) meaning that participation in the trial is unlikely to delay access to standard speech pathology intervention. Secondly, although the need for feedback in speech pathology intervention is accepted by clinicians and adopted into practice, the efficacy of feedback, let alone form or feedback, has not been established in the literature. Some of the treatment conditions in this protocol may expose children to non-standard feedback conditions but without an evidence base for standard practice, non-standard feedback conditions are no less appropriate to use. Thirdly, the treatment conditions proposed are based on sound principles of learning theory and speech pathology practice such that they may be of benefit and are highly unlikely to be of any harm. A final issue is that the control group could be perceived to be disadvantaged as they will not receive the experimental therapy nor traditional therapy during their participation. This is compensated by the fact that they will receive traditional therapy following their participation in the study.

### Feasibility and pilot study

We have a working HTML5 / JavaScript prototype which provides a) a practice game with images and sounds for three minimal pairs; and b) a demonstration game with KR feedback for a correct response for one minimal pair (Bee/Pea). The prototype is loaded onto a server from where it enables communication from a mobile device operated by a “Wizard” to a device operated by a child. The “Wizard of Oz” application will build on this prototype.

Once the application is developed, each option in the game will be tested in the Swinburne University BabyLab “Wizard of Oz” setting by the research team, including at least two speech pathologists. Once any revisions to the application are complete, the testing process will be repeated with four children aged four to six.

### Recruitment of participants

Potential participants will be identified from the Australian Catholic University Speech Pathology in Schools program in Melbourne. This program operates in several Catholic Education Primary Schools in Melbourne. Parents of clients of this service who are identified as having a phonological delay or disorder will be provided with an information sheet about the study. Parents who wish for their children to participate will be required to contact the staff of the study. Additional participants will be recruited through advertisement in kindergartens, day-care centers and playgroups.

Stratified random sampling based on severity of speech disorder and will be used to allocate children to experimental conditions. This process will ensure that each condition includes similar proportions of children with either a mild, moderate or severe phonological disorder. Due to the nature of the intervention, participants and therapists cannot be blinded to the experimental condition. However, pre- and post-test assessments of speech production will be carried out by a researcher/therapist blind to the assigned condition.

## Proposed analysis

The assessments of speech production will be transcribed to an Excel spreadsheet. Statistical analysis will be performed using the Statistical Package for the Social Sciences (IBM SPSS Statistics 24).

The key question to be addressed in this research is: How does feedback type during speech therapy session influence children's production performance. As such, the key dependent variable under analysis will be PCC, phonological process percentage of occurrence and percentage accuracy of trials. Published data of PCC in children's development (e.g., Dodd et al., 2002) suggest that this variable to be normally distributed. As such we intend to perform a full-factorial regression analysis on PCC with feedback condition, number of trials completed, and child age (4, 5, or 6 years) and severity as predictor variables. The full-factorial nature of this analysis will allow us to determine how the effects of condition and trials completed independently predict PCC and the extent to which these factors interact in their effects on PCC between pretest and post-test. For example, we may find that with certain types of feedback, the number of trials completed has a much greater effect than with other types of feedback. Similarly, we may find that some feedback types have a greater effect on performance for younger or older children. These analyses will also be completed with phonological process percentage of occurance at pretest and post-test and percentage accuracy of trials as a dependant variables

We will also conduct a Generalized Linear Model Analysis assuming a binomial distribution for the number of correct trials post “intervention” within a total of *n* trials where *n* may differ between children. The analysis will control for pre “intervention” language skills, age, and gender. Marginal means will be compared for the five groups using a Bonferroni correction for multiple comparisons. This analysis could assist in distinguishing the effects of real-world simulation vs. verification feedback and the presence of articulation guidance.

Motivation to persevere could be considered a variable that mediates the impact of type of feedback. Consequently, we will also perform a one-way ANOVA to determine how feedback condition influences motivation as measured by the number of trials and the duration of game play.

A G-Power power analysis suggested a total sample size of 150 in order to detect a moderate to large effect size (*f* = 0.32) with 5% significance and 80% power. Our recruitment plan of 30 participants in each of our 5 conditions is consistent with the G-Power analysis suggestion to avoid type II error.

## Anticipated results

With respect to research question 1, this study aims to investigate the effectiveness of four different types of feedback in improving speech sounds in young children while using a multimedia platform. Each of the three theoretical frameworks for learning discussed in the Background section predict that KR, the prevalent feedback in speech learning applications, will be the least effective because it does not contain information to assist learners in improving their speech production (Shute, 2008). The conversational framework for multimedia learning predicts that intrinsic feedback will be more effective than KR feedback because it is most likely to resemble real-world speech learning experiences which can be built upon (Laurillard, 2002). This framework predicts that the most effective feedback overall will be intrinsic combined with topic contingent KP because children will be able to elaborate and refine knowledge gleaned from real-world experiences with lessons learned during speech pathologist/learner dialogue (Laurillard, 2002). Within the context of novice-expert learning (Mason and Bruning, 2001; Moreno, 2004) children who are more responsive to therapy (influenced by factors such as age and severity of disorder) might benefit from KR combined with topic contingent KP, possibly because they could benefit from smaller amounts of more focused feedback. This combination is also prevalent in speech pathology practice.

With respect to research question 2, the prediction that the two conditions with intrinsic feedback will be the most effective in improving speech production is accompanied by the prediction that these two conditions will also be the most motivating because they will be situated in natural and social scenarios that could be more visually appealing, possibly more challenging (Mayer, 2014b) while offering greater autonomy and relevance (Ryan et al., 2006) and consequently should encourage more trials and longer gameplay. We also anticipate that other features of the respective feedback types will emerge from the analysis to suggest variables that will improve their effectiveness.

## Conclusion

It is difficult to find evidence of the efficacy of existing mobile applications to assist young children with developmental speech sound delay. This might be because they generally do not provide informative feedback about the sounds being made by the child. Considerable resources are required to develop mobile multimedia applications. By using a human “Wizard” to provide feedback about the speech sounds that children make while playing a game on a mobile device, evidence for effective feedback will be gathered before substantial development costs are incurred. The results of this experiment will inform the development of a Virtual Speech Therapist that provides pedagogically and clinically sound feedback to assist speech development in young children. The results could also inform other types of language learning.

## Author contributions

All authors contributed to conception and design of the protocol. All authors except HA wrote sections of the manuscript, read and approved the submitted version. WD and KF contributed to the illustrations and manuscript revision; WD commissioned and tested the prototype; HA contributed to the app review.

### Conflict of interest statement

The authors declare that the research was conducted in the absence of any commercial or financial relationships that could be construed as a potential conflict of interest. The reviewers and handling Editor declared their shared affiliation.

## References

[B1] Allen M. M. (2013). Intervention efficacy and intensity for children with speech sound disorder. J. Speech Lang. Hear. Res. 56, 865–877. 10.1044/1092-4388(2012/11-0076) 23275415

[B2] Baker E. (2010). Minimal pair intervention, in Interventions for Speech Sound Disorders in Children, eds Williams A. L. McLeod S. McCauley R. J. (Baltimore, MD: Paul H. Brookes Publishing Co), 41–72.

[B3] Baker E. (2012). Optimal intervention intensity. Int. J. Speech Lang. Pathol. 14, 401–409. 10.3109/17549507.2012.700323 22916999

[B4] Boyle E. A. Hainey T. Connolly T. M. Gray G. Earp J. Ott M. (2016). An update to the systematic literature review of empirical evidence of the impacts and outcomes of computer games and serious games. Comput. Educ. 94, 178–192. 10.1016/j.compedu.2015.11.003

[B5] Butler A. C. Godbole N. Marsh E. J. (2013). Explanation feedback is better than correct answer feedback for promoting transfer of learning. J. Educ. Psychol. 105, 290–298. 10.1037/a0031026

[B6] Clark R. C. Mayer R. E. (2016). E-Learning and the Science of Instruction Proven Guidelines for Consumers and Designers of Multimedia Learning. 4th Edn Hoboken, NJ: Wiley.

[B7] Community Affairs References Committee (2014). Prevalence of Different Types of Speech, Language and Communication Disorders and Speech Pathology Services in Australia. Canberra, ACT: Senate Community Affairs Committee Secretariat.

[B8] Connolly T. M. Boyle E. A. MacArthur E. Hainey T. Boyle J. M. (2012). A systematic literature review of empirical evidence on computer games and serious games. Comp. Amp. Educ. 59, 661–686. 10.1016/j.compedu.2012.03.004

[B9] Corbalan G. Kester L. van Merriënboer J. J. G. (2009). Dynamic task selection: effects of feedback and learner control on efficiency and motivation. Learn. Instr. 19, 455–465. 10.1016/j.learninstruc.2008.07.002

[B10] Crosbie S. Holm A. Dodd B. (2005). Intervention for children with severe speech disorder: a comparison of two approaches. Intl. J. Lang. Commun. Disord. 40, 467–491. 10.1080/13682820500126049 16195201

[B11] Deci E. Koestner R. Ryan R. M. (1999). A meta-analytic review of experiments examining the effects of extrinisc rewards on intrinsic motivation. Psychol. Bull. 125, 627–668. 10589297 10.1037/0033-2909.125.6.627

[B12] Dodd B. (2005). Children with speech disorder: defining the problem, in Differential Diagnosis and Treatment of Children With Speech Disorder, ed Dodd B. (London: Whurr), 2–23.

[B13] Dodd B. (2014). Differential diagnosis of pediatric speech sound disorder. Curr. Dev. Disord. Rep. 1, 189–196. 10.1007/s40474-014-0017-3

[B14] Dodd B. Hau Z. Crosbie S. Holm A. Ozanne A. (2002). Diagnostic Evaluation of Articulation and Phonology. London: Pearson.

[B15] Engwall O. Bälter O. (2008). Pronunciation feedback from real and virtual language teachers. Comp. Assist. Lang. Learn. 20, 235–262. 10.1080/09588220701489507

[B16] Engwall O. Balter O. Oster A.-M. Kjellstrom H. (2006). Designing the user interface of the computer-based speech training system ARTUR based on early user tests. Behav. Inf. Technol. 25, 353–365. 10.1080/01449290600636702

[B17] Hainey T. Connolly T. M. Boyle E. A. Wilson A. Razak A. (2016). A systematic literature review of games-based learning empirical evidence in primary education. Comput. Educ. 102, 202–223. 10.1016/j.compedu.2016.09.001

[B18] Harks B. Rakoczy K. Hattie J. Besser M. Klieme E. (2014). The effects of feedback on achievement, interest and self-evaluation: the role of feedback's perceived usefulness. Educ. Psychol. 34, 269–290. 10.1080/01443410.2013.785384

[B19] Hewlet N. (1990). Process of development and production, in Developmental Speech Disorders, ed Grunwell P. (Edinburgh: Churchill Livingstone), 15–38.

[B20] Huber B. Tarasuik J. Antoniou M. N. Garrett C. Bowe S. J. Kaufman J. (2016). Young children's transfer of learning from a touchscreen device. Comput. Human Behav. 56, 56–64. 10.1016/j.chb.2015.11.010

[B21] Kaipa R. Peterson A. M. (2016). A systematic review of treatment intensity in speech disorders. Int. J. Speech Lang. Pathol. 18, 507–520. 10.3109/17549507.2015.1126640 27063688

[B22] Ke F. (2014). An implementation of design-based learning through creating educational computer games: a case study on mathematics learning during design and computing. Comput. Educ. 73, 26–39. 10.1016/j.compedu.2013.12.010

[B23] Laurillard D. (2002). Rethinking University Teaching: A Conversational Framework for the Effective Use of Learning Technologies, 2nd Edn New York, NY: Routledge.

[B24] Maas E. Robin D. A. Hula S. N. Freedman S. E. Wulf G. Ballard K. J.. (2008). Principles of motor learning in treatment of motor speech disorders. Am. J. Speech Lang. Pathol. 17, 277–298. 10.1044/1058-0360(2008/025) 18663111

[B25] Mason B. J. Bruning R. H. (2001). Providing Feedback in Computer-based Instruction: What the Research Tells Us. CLASS Research Report No. 9.

[B26] Massaro D. W. Light J. (2004). Using visible speech to train perception and production of speech for individuals with hearing loss. J. Speech Lang. Hear. Res. 47, 304–320. 10.1044/1092-4388(2004/025) 15157132

[B27] Masso S. McCabe P. Baker E. (2014). How do children with phonological impairment respond to requests for clarification containing polysyllables? Child Lang. Teach. Ther. 30, 367–382. 10.1177/0265659013516330

[B28] Mayer R. E. (2005). Cognitive Theory of Multimedia Learning. Cambridge; NewYork, NY: Cambridge University Press.

[B29] Mayer R. E. (2014a). Computer Games for Learning: an Evidence-Based Approach. Cambridge, MA: The MIT Press.

[B30] Mayer R. E. (2014b). Incorporating motivation into multimedia learning. Learn. Instr. 29, 171–173. 10.1016/j.learninstruc.2013.04.003

[B31] Merchant Z. Goetz E. T. Cifuentes L. Keeney-Kennicutt W. Davis T. J. (2014). Effectiveness of virtual reality-based instruction on students' learning outcomes in K-12 and higher education: a meta-analysis. Comput. Educ. 70, 29–40. 10.1016/j.compedu.2013.07.033

[B32] Moreno R. (2004). Decreasing cognitive load for novice students: effects of explanatory versus corrective feedback in discovery-based multimedia. Instr. Sci. 32, 99–113. 10.1023/B:TRUC.0000021811.66966.1d

[B33] Narciss S. (2017). Conditions and effects of feedback viewed through the lens of the interactive tutoring feedback model, in Scaling Up Assessment for Learning in Higher Education, eds Bridges S. M. Carless D. C. Yuk C. K. Glofcheski R. (Singapore: Springer), 173–188.

[B34] Narciss S. Huth K. (2004). How to design informative tutoring feedback for multimedia learning, in Instructional Design for Multimedia Learning, eds Niegemann H. M. Leutner D. Brunken R. (Munster; New York, NY: Waxmann), 181–195.

[B35] Narciss S. Sosnovsky S. Schnaubert L. Andrès E. Eichelmann A. Goguadze G. (2014). Exploring feedback and student characteristics relevant for personalizing feedback strategies. Comput. Educ. 71, 56–76. 10.1016/j.compedu.2013.09.011

[B36] Hodson B. (2010) Evaluating and Enhancing Children's Phonological Systems: Research and Theory to Practice. Wichita, KS: Phonocomp Publishers.

[B37] Redford M. A. Diehl R. L. (1999). The relative perceptual distinctiveness of initial and final consonants in CVC syllables. J. Acoust. Soc. Am. 106, 1555–1565. 10.1121/1.427152 10489711

[B38] Renals S. King S. (2010). Automatic Speech Recognition the Handbook of Phonetic Sciences, 2nd Edn Oxford: Blackwell Publishing Ltd 804–838

[B39] Ryan R. M. Rigby C. S. Przybylski A. (2006). The motivational pull of video games: a self-determination theory approach. Motiv. Emot. 30, 347–363. 10.1007/s11031-006-9051-8

[B40] Semel E. M. Wiig E. H. Secord W. A. Hannan T. (2006). Clinical evaluation of language fundamentals, in CELF-4: Australian, 4th Edn., Australian Standardised Edition. ed PsychCorp (Sydney, NSW: Pearson Clinical and Talent Assessment).

[B41] Shute V. J. (2008). Focus on formative feedback. Rev. Educ. Res. 78, 153–189. 10.3102/0034654307313795

[B42] Topping K. Dekhinet R. Zeedyk S. (2013). Parent–infant interaction and children's language development. Educ. Psychol. 33, 391–426. 10.1080/01443410.2012.744159

[B43] van Doremalen J. Boves L. Colpaert J. Cucchiarini C. Strik H. (2016). Evaluating automatic speech recognition-based language learning systems: a case study. Comp. Assisted Lang. Learn. 29, 833–851. 10.1080/09588221.2016.1167090

[B44] van Heuven W. J. Mandera P. Keuleers E. Brysbaert M. (2014). SUBTLEX-UK: a new and improved wordfrequency database for British English. Q. J. Exp. Psychol. 67. 1176–1190. 10.1080/17470218.2013.850521 24417251

[B45] Weiner F. F. Ostrowski A. A. (1979). Effects of listener uncertainty on articulatory consistency. J. Speech Hear. Disord. 25, 300–309. 10.1044/jshd.4404.487 513671

[B46] Wiig E. H. Semel E. M. Secord W. Carstairs J. (2006). CELF Preschool 2 : Australian and New Zealand Clinical Evaluation of Language Fundamentals, Preschool Clinical Evaluation of Language Fundamentals, Preschool, 2nd Edn Sydney, NSW: Pearson Clinical and Talent Assessment; PsychCorp.

[B47] Williams A. L. (2012). Intensity in phonological intervention: is there a prescribed amount? Int. J. Speech Lang. Pathol. 14, 456–461. 10.3109/17549507.2012.688866 22686582

[B48] Yont K. Hewitt L. Miccio A. (2000). A coding system for describing conversational breakdowns in preschool children. Am. J. Speech – Lang. Pathol. 9, 300–309. 10.1044/1058-0360.0904.300

